# Pulmonary aspergilloma on transthoracic ultrasound

**DOI:** 10.1007/s15010-021-01589-7

**Published:** 2021-03-09

**Authors:** Lisa C. Ruby, Rajagopal Kadavigere, Shubha Sheshadri, Kavitha Saravu, Sabine Bélard

**Affiliations:** 1 Department of Pediatric Respiratory Medicine, Immunology and Critical Care Medicine, Charité-Universitätsmedizin Berlin, Berlin, Germany; 2 Department of Radiodiagnosis, Kasturba Medical College, Manipal Academy of Higher Education, Manipal, India; 3 Department of Medicine, Kasturba Medical College, Manipal Academy of Higher Education, Manipal, India; 4 Department of Infectious Diseases, Kasturba Medical College, Manipal Academy of Higher Education, Manipal, India; 5 Manipal Center for Infectious Diseases, Prasanna School of Public Health, Manipal Academy of Higher Education, Manipal, India; 6 Berlin Institute of Health, Berlin, Germany

**Keywords:** Ultrasound, Aspergilloma, Tuberculosis, Lung

## Abstract

**Purpose:**

Pulmonary aspergilloma affects immunocompromised patients but is also a recurrent condition in patients previously treated for pulmonary tuberculosis.

**Methods and Results:**

We report the case of a 45-year-old patient with a history of cured pulmonary tuberculosis 15 years earlier in whom we visualized pulmonary aspergilloma by transthoracic lung sonography. Sonography of pulmonary aspergilloma demonstrated an oval cavity with hypoechoic contents and an irregular border, measuring a diameter of 4.7 cm; inside the lesion, a roundish structure with an anechoic rim was discernable.

**Conclusions:**

The sonographic findings corresponded to chest X-ray and computed tomography imaging in this patient and to previously reported sonographic characteristics of mycotic abscesses in other organs. Lung ultrasound may be a tool to identify pulmonary aspergilloma, especially as a point-of-care imaging tool and where other imaging modalities are inaccessible.

## Background

A 45-year-old male non-smoker with a history of cured pulmonary tuberculosis 15 years earlier and without known co-morbidities presented with fatigue, chronic cough, dyspnea on exertion, and weight loss for 3 months. One month before his presentation, he reported having developed a fever for 1 week and having received outpatient treatment with antibiotics for 20 days without clinical improvement and progression of weight loss.

On admission, the patient was afebrile and in a poor nutritional status with a BMI of 14.5. Physical examination revealed bronchial breath sounds over the left upper lobe and bilateral inspiratory coarse crepitations. Oxygen saturation was 88% on room air. Sputum microscopy and culture were negative for respiratory bacteriology. Three sputa were negative for acid-fast bacilli on microscopy and negative on Xpert^®^. Complete blood count showed anemia with a hemoglobin of 10.0 g/dl but was otherwise normal (leukocytes 6.4 × 103/µl; 73.1% neutrophils, 18.6% lymphocytes, 6.6% monocytes, 1.3% eosinophils, 0.4% basophils; platelets 349.0 × 103/µl). The HIV test was negative and CRP at 44.81 mg/l.

**Fig. 1:**
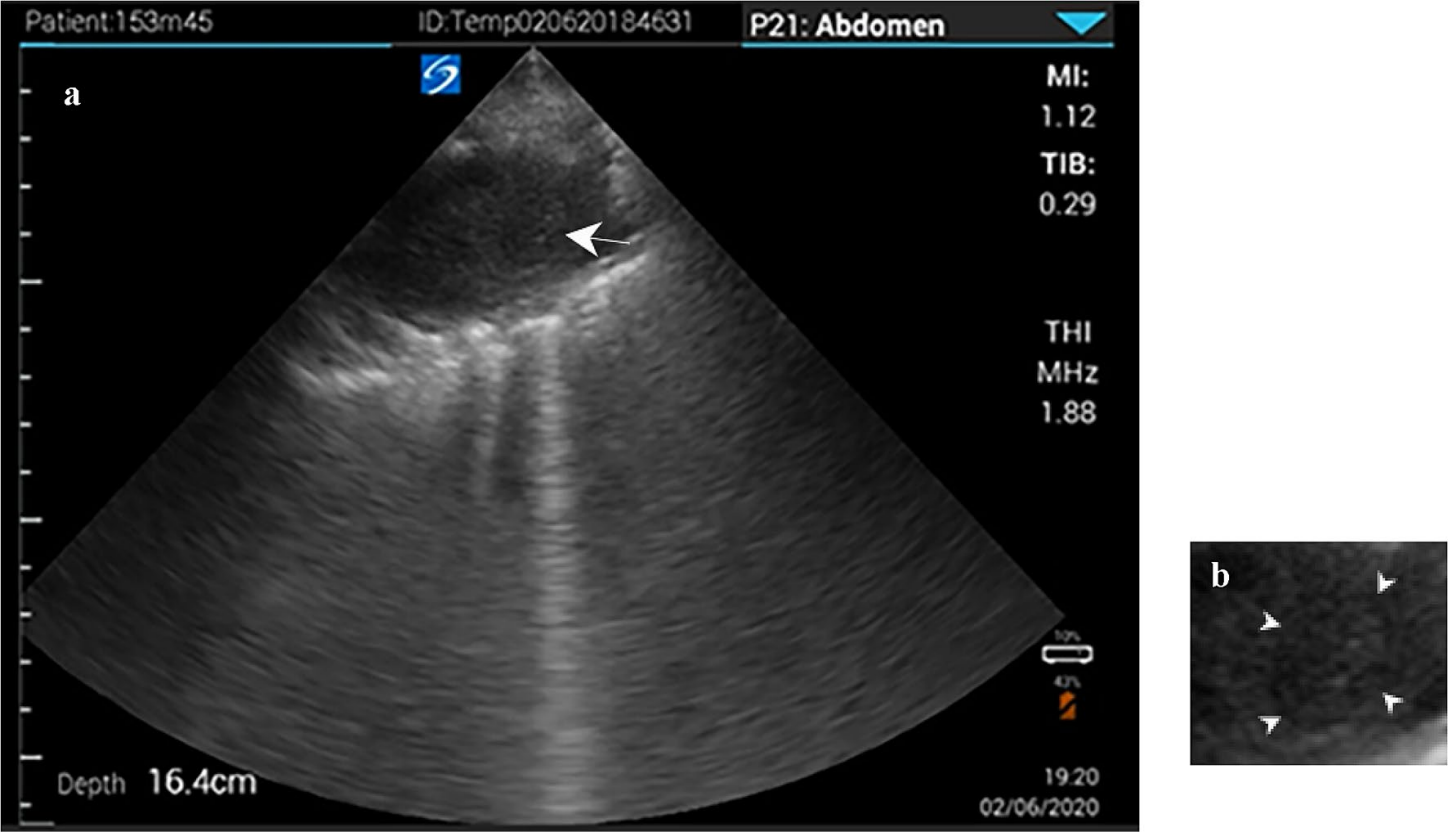
**a** Sonographic image with phased array probe shows oval cavity with irregular border and hypoechoic contents, the arrow marks a roundish structure with an anechoic rim. **b** Roundish structure, the arrowheads mark the anechoic rim

Point-of-care lung ultrasound was performed as part of a diagnostic work-up for febrile respiratory disease with a handhold tablet device (iViz SonoSite) using a linear and sector probe. Ultrasound showed a markedly irregular pleural line, particularly in anterosuperior regions bilaterally. Multiple small bilateral subpleural consolidations as well as multiple larger consolidations were seen with a maximum size of 2.6 cm. Impressively, an oval cavity with irregular border measuring a diameter of 4.7 cm with hypoechoic contents was seen on the anterolateral aspect on the left side. Inside the lesion, a roundish structure with an anechoic rim was noted (Fig. 1a–b).

**Fig. 2:**
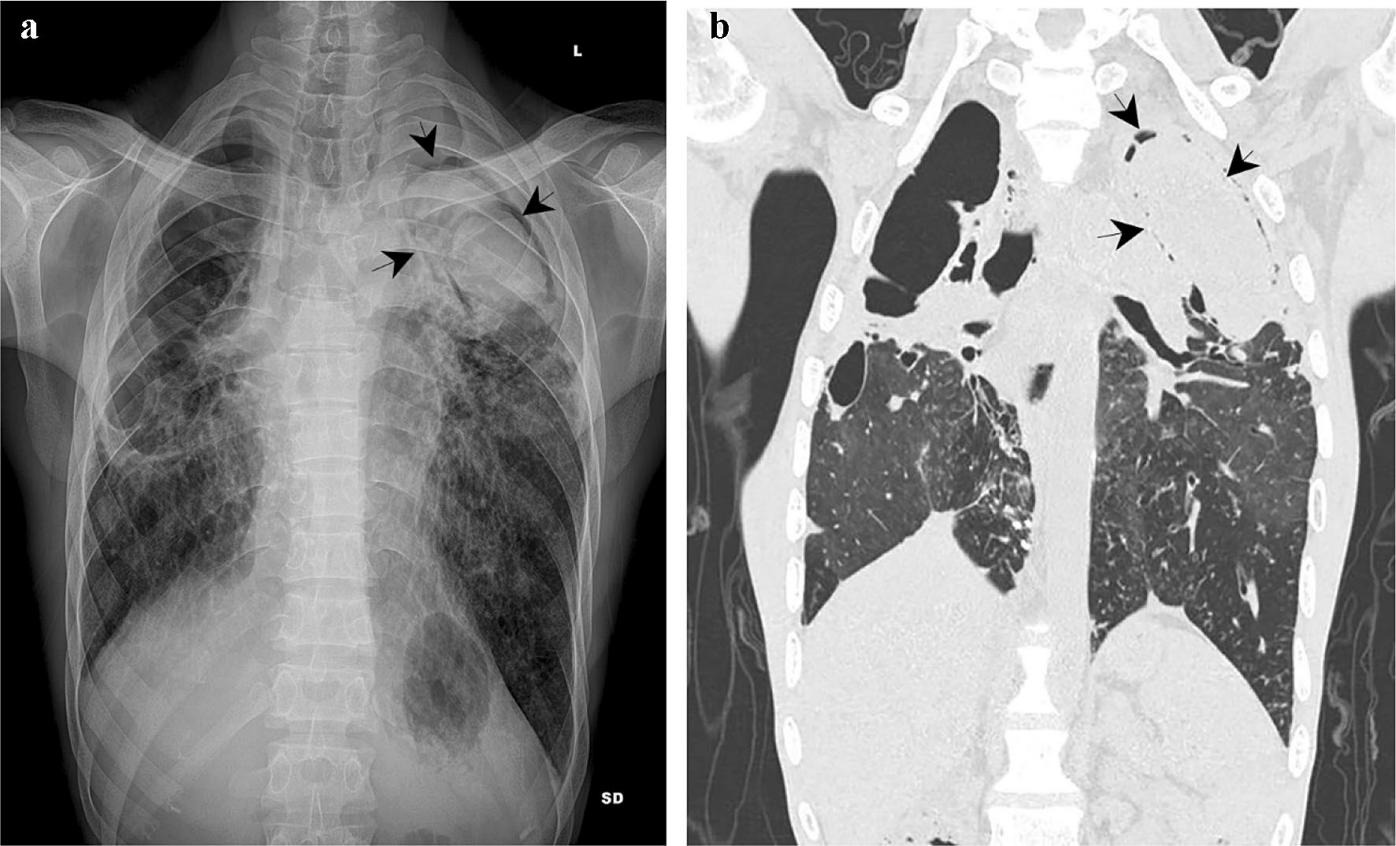
Posterior-anterior radiograph of the chest (**a**) and coronal CT reformation (**b**) shows fibrocavitary lesions in bilateral upper lobes. Left upper lobe cavity shows dependant intracavitary opacity with air crescent (arrow) in keeping with aspergilloma

On chest X-ray a tuberculous cavity with aspergilloma was reported, thereby matching the sonographic finding (Fig. 2a). Contrast-enhanced computed tomography (CECT) of the thorax revealed complete destruction of lung parenchyma with fibrosis, tubular-cystic bronchiectasis, cavity formation, and cicatrization in both lungs in line with chronic sequelae of pulmonary tuberculosis. Additionally, signs of secondary infection were seen presenting as multiple nodules of varying sizes with surrounding ground-glass attenuation predominantly in the posterobasal segment of the left lung. In line with the cavity seen on ultrasound and CXR, a non-enhancing soft tissue density with few calcified foci surrounded by an air crescent was seen in a cavity in the left upper zone measuring 6 × 4.5 × 6 cm (Fig. 2b); an additional even larger cavity of 10 × 10 × 13 cm was seen in the right upper zone. Echocardiography diagnosed severe pulmonary hypertension. Bronchoscopy detected increased mucoid secretions in the left upper lobe. Bronchial washing was sent for smear microscopy and showed few singly scattered endobronchial cells, squamous cells and anthracotic pigment laden macrophages in a background of neutrophils and lymphocytes. An aerobic culture of bronchoalveolar lavage fluid allowed isolation of *Pseudomonas aeruginosa* after discharge of the patient. An ELISA for Aspergillus Galactomannan Antigen was positive in broncho alveolar lavage fluid.

The patient was consequently diagnosed with aspergilloma and angioinvasive aspergillosis as well as post TB sequelae. Cardiothoracic surgery was consulted and conservative management consisting of watch and wait advised. Sildenafil was started for pulmonary hypertension. The patient passed away 1 month after his visit to the hospital without further consultations or treatments. The patient’s relatives consented to the publication of this case report.

## Discussion

Diseases by aspergillus species mostly affect immunocompromised patients with a broad clinical spectrum that comprises a variety of manifestations even within the lungs. Local pulmonary disease can present as aspergilloma, representing mycelial ball growing in damaged lung areas such as cavities [1]. In semi-invasive disease aspergillomas occur concurrently to progressive fibrosis with minor invasion taking place. Distinct sub-entities have been suggested but are summarized by the term “chronic pulmonary aspergillosis” (CPA) [2] with around 25% of patients with CPA also having an aspergilloma [3].

Chronic pulmonary aspergillosis (CPA) affects an estimated 3 million people worldwide [4]. Noteworthy, WHO reports that approximately one third of patients diagnosed with CPA was previously treated for pulmonary tuberculosis (PTB) [3]. Initial clinical presentation of CPA and PTB are often alike. In resource-limited facilities testing for immunoglobulin G (IgG) antibodies against *A. fumigatus* may often not be available, and thus CPA may often be undiagnosed or misdiagnosed as “smear-negative PTB” and/or “relapse” of TB in endemic settings [5]. Post-TB lung disease (PTLD) is responsible for a notable number of chronic lung diseases worldwide [6] and its wide-ranging consequences are yet to be understood fully to improve TB treatment outcomes [7].

Lung ultrasound (LUS) has become an established diagnostic imaging modality for pulmonary conditions in recent years. LUS became a standard tool in emergency medicine for timely diagnosis of pneumothorax [8] as well as ARDS [9] and has proven useful as a radiation-free imaging modality with high test accuracy for diagnosis of pneumonia in childhood [10]. Lung ultrasound was even found suitable to differentiate bacterial from viral etiology of community-acquired pneumonia in children [11].

Reports on the visualization of pulmonary aspergilloma by ultrasound are limited. A clinical report from 2012 describes color Doppler sonography (CDS) of pulmonary aspergillosis in infants with chronic granulomatous disease with detection of systemic arterial feeders to the pleural-based lesions and sonographic findings of aspergillosis reported as “crescent” or “halo” sign, which, interestingly, were not visible on CT [12]. A publication on endobronchial ultrasound from 2016 reports a “central annular image in an ill-defined hypoechoic paraoesophageal lesion” as possibly sonographic characteristic of pulmonary aspergillosis [13]. A similar sonographic characteristic described as the “fluid rim sign” was reported in a case report of musculoskeletal aspergillosis [14]. For hepatosplenic mycotic abscesses the “wheel within wheel” appearance is described as a specific finding on ultrasound [14].

Using a phased array probe on the left upper zone we obtained transthoracic ultrasound images of a comparable composition with a central roundish structure and a fine anechoic rim (Fig. 1a–b), adding to the hypothesis that this might be a characteristic finding of pulmonary aspergillosis. Visualization of the aspergilloma by lung ultrasound was only possible as the cavity reached the pleura and the mycelial ball almost filled the cavity leaving very little air-filled space within the cavity to interfere with ultrasound. In contrary, the other, even larger cavity in the right upper zone was not clearly discernable on lung ultrasound. Fibrotic margins of this cavity, which were immediately adjacent to the pleura, were detectable as noteworthy pleural irregularities, yet, this massive air-filled space (Fig. 2) could not be comprehensively visualized on ultrasound to adequately detect the lesion as cavity.

Tuberculous cavities on ultrasound have been well described by Agostinis et al. as presenting as roundish anechoic or hypoechoic areas within solid consolidations that have thick and irregular walls and posterior enhancement [15]. Detection rates of cavities on LUS are however low [16, 17], most likely due to air-filled lung between cavity and pleura which impedes visualization of underlying findings. The “crescent” sign has not been described for tuberculous cavities and may therefore be a sonographic feature for differentiating mycotic from tuberculous cavities.

Ultrasound at the point-of-care has developed into a relatively cheap, portable and radiation-free diagnostic tool to augment physical examination, especially where resources are scarce [10]. The value of LUS is being studied for pulmonary TB as low- and middle-income countries often bear a high burden of tuberculosis (TB) and access to standard imaging may be limited [15, 18–20]. LUS detects features of pulmonary TB including cavitary or miliary presentation; however, the full discriminatory power of LUS to differentiate PTB from other pulmonary conditions remains to be established. The potential of LUS is an expanding area of research and it might also be worth to further investigate possible distinctive US patterns for different etiologies of pulmonary diseases including detection of pulmonary aspergilloma. However, lung ultrasound does never allow to rule out focal lung pathology because if there is normal lung in between pleura and pathology a characteristic image will not be obtained; for focal pulmonary pathology lung ultrasound may therefore be relevant as a “rule in test” but not as a “rule out test”.

More studies with an imaging comparator such as computed tomography are needed to further evaluate the significance of LUS on cavity detection. We would like to add our findings to the current evidence on this topic and stress that LUS on pulmonary aspergilloma is an interesting area to investigate further to explore US as a novel diagnostic option as timely, radiation-free, and cost-efficient imaging.
